# The Reduction of Distress Using Therapeutic Geothermal Water Procedures in a Randomized Controlled Clinical Trial

**DOI:** 10.1155/2015/749417

**Published:** 2015-03-19

**Authors:** Lolita Rapolienė, Artūras Razbadauskas, Antanas Jurgelėnas

**Affiliations:** ^1^Seamen's Health Care Center, Taikos 46, LT-91213 Klaipėda, Lithuania; ^2^Klaipėda University, Herkaus Manto Gatvė 84, LT-92294 Klaipėda, Lithuania; ^3^State Research Institute Centre for Innovative Medicine, Žygimantų 9, LT-01102 Vilnius, Lithuania

## Abstract

Stress is an element of each human's life and an indicator of its quality. Thermal mineral waters have been used empirically for the treatment of different diseases for centuries. *Aim of the Study*. To investigate the effects of highly mineralised geothermal water balneotherapy on distress and health risk. *Methodology*. A randomized controlled clinical trial was performed with 130 seafarers: 65 underwent 2 weeks of balneotherapy with 108 g/L full-mineralisation bath treatment; the others were in control group. The effect of distress was measured using the General Symptoms Distress Scale. Factorial and logistic regression analyses were used for statistical analysis. *Results*. A significant positive effect on distress (*P* < 0.001) was established after 2 weeks of treatment: the number of stress symptoms declined by 60%, while the intensity of stress symptoms reduced by 41%, and the control improved by 32%. Health risks caused by distress were reduced, and resources increased, whereas the probability of general health risk decreased by 18% (*P* = 0.01). *Conclusion*. Balneotherapy with highly mineralised geothermal water reduces distress, by reducing the health risk posed by distress by 26%, increasing the health resources by 11%, and reducing probability of general health risk by 18%. Balneotherapy is an effective preventive tool and can take a significant place in integrative medicine.

## 1. Introduction

**Figure 1:**
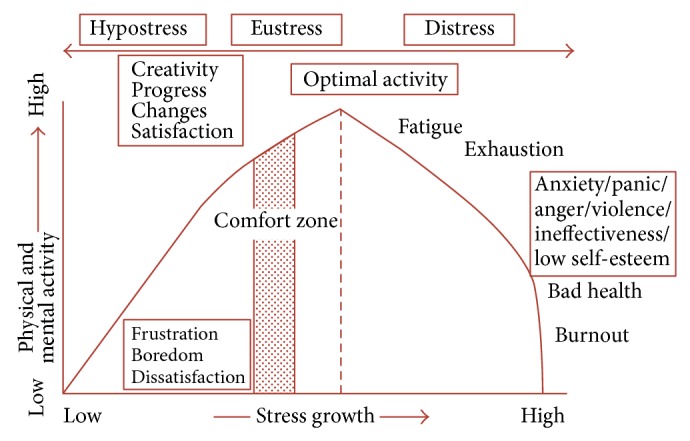
Human response to stress curve (^*^according to Nixon P: Practitioner 1979, Yerkes RM, Dodson JD).

Stress is an important element of our lives which depends on social, economic, psychological, physical, and intellectual aspects of development and change and is an index of the quality of life [1]. The presence of some stress should prompt changes, progress, and creativity, while the high levels of stress or long-term stress, if uncontrolled, causes health problems, reduces the capacity to work, and diminishes the quality of life (Figure 1) [1–4]. Stress is widespread: in 2012, 87% of adults in the USA and Great Britain agreed that stress is a serious health problem; 63% had acquaintances who had faced health consequences due to stress, and more than 4 out of 10 experienced stress themselves [4]; in Canada more than 6 employees out of 10 who indicated great levels of stress consider that the main reason of stress is work (2010) [5]. There is cumulating evidence that socioeconomic status (disparities in income, education, occupation, etc.) accounts for substantial variance in all-cause and disease-specific morbidity and mortality rates and for prevalent psychopathologies of mood [1, 6]. Stress is widespread among health and education system workers, other civil service workers, and seafarers [7–10]. According to studies, 28.3 to 63.5% of seafarers experience stress at work [11]. It is influenced by the lack of sleep, bad quality of sleep, long working hours, extended work, work not according to biorhythms, lack of rest, big load, noise, heat, vibration, restricted movement, dehydration, time zone and climate change, absence of medical professionals, irregular sexual life, social exclusion, and other factors [11–13].

Individual reactions to stress may display in emotional, cognitive, pathological, physiological, and behavioural symptoms. Once in a stressful situation, the body's different systems: skin, respiratory, digestive, endocrine, cardiovascular, and nervous systems, begin to react [1, 4, 14–16]. These processes, which are a part of systematic efforts, are activated in order to restore an individual's homeostasis [17].

A survey of stress in America revealed that 31% of people are doing a poor/fair job at recovering fully or recharging after they have been stressed out [18]. Currently, there are no absolutely efficient and optimal stress removal methods. A wide range of interventions are proposed: primary psychological help after acute stress, search for the existing psychosocial stressors (situations, supportive family members, and community service), stress reduction (breathing techniques, progressive muscle relaxation, cultural equivalents, and hobbies), social assistance (personal strength and skills, stimulation to return to the social environment, emotional support, and warning for damaging decisions), reduction of insomnia, hyperventilation, sensorimotor dissociation (yoga, meditation, reiki, and electrotherapy), regulation of work and rest schedule, psychological training, cognitive behavioural therapy, eye movement desensitisation and reprocessing, reflexology, relaxing therapies, massages, acupuncture, aromatherapy, music therapy, balneotherapy, medication, and so forth [19–25].

Balneotherapy (Latin* balneum*, bath) has been defined as the use of natural mineral waters, natural peloids and mud, and natural sources of different gases (CO_2_, H_2_S, and Rn) for medical purposes such as prevention, treatment, and rehabilitation [26]. Thermal mineral waters have been used empirically for the treatment of different diseases for centuries [25, 27]. In recent decades in the world, this branch of medicine is experiencing a major transformation due to scientific evidences of the effects of mineral water, which restores confidence in the old therapeutic procedures and eliminates prejudice.

The essence of balneotherapy effects is local changes caused by the direct influence of mechanical, thermal, and chemical factors through the skin and mucous membranes and complex adjustment reactions as a result of neuroreflexive, humoral mechanisms, caused by stimulation of mechano-, thermo-, baro-, and chemoreceptors by biochemical active substances during the balneoprocedure [28]. Due to the thermal effect, blood flow in the skin, subcutaneous tissue, muscles, and organs is increasing, increasing also the production of physiologically active substances that act on the nervous and endocrine systems, promote angiogenesis, and reduce ischemia [29, 30]. Hydrostatic pressure and buoyancy affect muscle tone and joint mobility, reducing the load and decreasing pain intensity. Redistribution of blood reduces peripheral resistance and increases diuresis and cerebral blood flow, which can improve brain functions, including cognition and memory [29, 31, 32] and stress reactivity, coping, and recovery processes [1]. Immersion in water affects the autonomic nervous system by reducing sympathetic power, thus reducing anxiety [31]. The transport of chemicals through the skin is a complex process. Venier, Larese, and Filon's studies show that metallic ions can easily permeate the skin (nickel, cobalt, and chromium) [33]. Human skin is permeable to Mg, Ca, K, Cu, Br, Rb, Ca, Fe, Pb, Cd, and Zn ions [34–37] and sulfates and nitrate anions [35]. Factors influencing percutaneous absorption through the skin include (1) physicochemical properties of the test compound, (2) skin properties and metabolism, (3) dose and volume of test substance, and (4) duration of exposure [38]. Sweating and skin hydration have been reported to increase dermal absorption. Over the decades, a large number of data have been generated on the percutaneous penetration of a wide range of chemicals, pesticides, cosmetics, and pharmaceuticals [38]. During balneotherapy studies, effects on hormones, cytokines, cell populations, C-reactive protein, haptoglobulin, substance P, matrix metalloproteinases, oxidative/antioxidant system, *β*-2-microglobulin, nitrogen oxide, insulin growth factor-1 (IGF1), transforming growth factor *β* (TGF-*β*) levels, opioid secretion, modification of SERT receptors were determined [39–43]. The overall effect of the application of balneofactors depends on their physical properties, as well as on the way of their implementation [25, 27, 28, 40]. Although the full mode of the effect of balneotherapy is still unclear, its efficacy appears confirmed by reviews [44, 45].

It was demonstrated that balneotherapy affects cardiovascular, musculoskeletal, endocrine, autonomic nervous, and other systems and brings positive results for public health [25, 26, 46, 47]. According to the results shown by studies, the improvement of clinical parameters is significantly greater with thermal mineral water than with tap water [48–53].

Natural mineral water is a general term applied to both spring and other underground continental waters (from deep-seated water wells). Due to the high temperature and high mineralisation, geothermal resources, such as hot springs or geothermal water from well, take an important place in the sectors of health and wellness [54]. About 25% of the direct use of geothermal water in the world is attributed to balneotherapy and bathing (2010) [54, 55].

According to the medical dictionary, health risk is a disease precursor associated with a higher than average morbidity or mortality rate. Health risk, as an integral phenomenon, consists of the deviation of the majority of an organism's functions from normal, which is provoked by many factors. One of most significant factors is distress [3, 17]. By applying probabilistic analysis to human health risk assessments, we can effectively characterize variability and uncertainty in risk, which can lead to enhanced site decision-making for intervention effectiveness. Quantifying and qualifying of integral risk, as integrated risk governance, are stressed in documents and publications [56–59]. We can account distress as a hazardous factor risk, and its health impact is also intervention impact on the change of health risk by using systemic approach where the health process development depends on positive and negative factors. Positive factors increase health recourses, while negative ones increase the risk. Depending on the health resources and risk profile, health development can take a balanced (normal), positive, or negative character [60, 61].

As balneotherapy affects the majority of the organism's functions positively including the stress-related ones, we can make the hypothesis that balneotherapy has an integral effect and can affect health risk positively.

*Aim of the study* was to investigate the effects of highly mineralised geothermal water balneotherapy on distress and health risk.

## 2. Materials and Methods

The clinical trial was carried out in Klaipėda Seamen's Hospital Maritime Medicine Centre, and in Klaipėda Seamen's Health Care Centre, Klaipeda, Lithuania (2012). This open-label randomized controlled trial was implemented in observance of the rules of good clinical practice; protocol was approved by the regional Research Ethics Committee. Participants were 130 male seafarers aged between 25 and 64 years, working at sea for more than 5 years. All subjects were informed about the purpose, conditions, and course of the study prior to inclusion and signed participants' agreement. Criteria for exclusion were acute organic neurological deficit, neoplastic or inflammatory lesion, decompensated cardiovascular disease, unstable metabolic disorders, febrilic infections, and cutaneous suppuration. The coding and randomisation of the respondents were applied to avoid subjective influences. The 130 studied participants were randomized into two groups: the geothermal water group (65) and the control (65) group. An individual who was not involved in the implementation of the study arranged the randomization according to the numbered series of prefilled envelopes.

65 participants underwent the course of 6 to 10 balneotherapy procedures, while the rest of the respondents were a part of the control group and did not receive any treatment. Balneotherapy was carried out on an outpatient on an everyday basis, for 5 days a week over a 2-week period, without changing their daily routines or going to work. The participants of the control group were not given any therapy and lived their usual life with no change in their daily routine or work attendance.

Balneotherapy procedure was as follows: the bathtub was filled with 200 liters of geothermal water. The participants had baths (immersing up to the armpits) for 15 minutes monitored by the trained personnel. Each participant was told to move slightly in the bathtub. After the baths, participants were recommended to gently dry the skin with a towel and not to shower for about one hour to prolong the effects of the procedure [25].

Geothermal water was naturally warm (34.6°C on average) with highly mineralised (108 g/L) geothermal Na-Cl-Ca-Mg-SO_4_ mineral water (pH 6.07) from Geoterma 2P (ID 25871) borehole (1135 m depth, lower Devonian layer, and mineral age of about 1 million years). Water composition can be expressed by the Kurlov formula (ekv/%):

(1) $$\begin{array}{c} \text{M}108.2\frac{\text{Cl}98}{\text{Na}64\text{Ca}24\text{Mg}12}. \end{array}$$

Volumetric activity of radon in the water was 29 ± 5 Bq/L. The water composition is shown in Table 1.

### 2.1. Study Outcome

Distress and health risk change after balneotherapy with geothermal water. Baseline and post-therapy (after 2 weeks) distress symptoms, their severity, and the management of them were measured by the self-assessment scale General Symptoms Distress Scale (GSDS) [29, 62]. No follow-up assessment was made because of participants' group specifics and unpredictable compliance during sea period. Distress was identified by marking in the survey the symptoms that the patients currently experienced out of 12 symptoms given, prioritizing them, marking how distressing each symptom is to them and how well they are able to manage their symptoms on a scale of 1 to 10.

The risk formed by distress was identified by latent factors consisting of the relationships' correlation between distress survey symptoms. Latent factors were calculated using factorial analysis. As the values of the factors vary from +3 to −3 on average, negative values were assigned to distress risk, while positive values were assigned to the resources.

### 2.2. Statistical Analysis

To describe the data, we calculated index averages and standard deviations (SD). To determine the accuracy and reliability of the statistical evaluation, we calculated confidence intervals with the confidence level of 0.95. Calculations were done using SPSS 21 statistical program. For comparisons, means before and after used paired-samples *t*-test. For comparisons means between geothermal and control groups used Student's *t*-test. To assess the quantitative symptoms, the arithmetic mean and its 95% confidence interval (CI) were calculated, while the logistic regression method was used to evaluate the interconnection of factors. The probability ratios (PR) and their 95% confidence intervals (CI) were calculated. The probability ratio is considered statistically reliable if the unit is not in the confidence interval of 95%. The factorial analysis method was used to search for common latent factors characterising distress and risk. The factorial positive and negative values and their percentile distribution were calculated and evaluated. The main components method was applied for determining the factors by applying the varimax method of the coordinates' rotation. The suitability of variables for factorial analysis was verified by Kaiser-Meyer-Olkin (KMO) criterion. Variables with generality less than 0.3 were not included into the factorial analysis. The factorial analysis evaluated the correlation between the symptoms. A common latent factor contained more information than any symptom under investigation.

## 3. Results

The groups of the respondents were similar in age, family status, period of service, and subjective assessment of health, as well as stress, pain, and fatigue intensity. A higher incidence of diseases, bad sleep quality, and alcohol use were observed in the geothermal group. Representatives of the control group had a higher body mass index (BMI), were taking more medications, and were smoking more frequently. Participants' characteristics are provided in Table 2.

Study results regarding the change of distress after 2 weeks were favourable to balneotherapy.

Assessment of the geothermal group using a GSDS revealed that, after two weeks, a statistically significant positive effect was established in all three distress elements (*P* < 0.001): the general amount of stress symptoms decreased by 2.52 (60%), and the intensity of stress symptoms declined by 2.16 (41%), while the stress management improved by 1.85 points (32%) (Table 3). The control group had no significant changes.

In the geothermal group, the distress symptoms significant for health risk were investigated using factorial analysis; latent factors forming distress risk were searched for. Twelve GSDS symptoms were used for analysing seafarers' distress; eleven symptoms met the conditions of the factorial analysis (the symptom of vomiting was not significant). Four latent factors of distress were investigated; they explained 57.22% of the total variance (Table 4).

The first factor connecting the three symptoms that covered the characteristics related to the central nervous system was attributed to the mental latent factor. It accounted for 15.75% of total variance; factor values ranged from −1.61 to 4.59. Positive values were determined for 32.7% of respondents. The second factor, that integrated the three symptoms, which partially characterised the digestive activity, was named dyspeptic. This distress latent factor accounted for 15.52% of total variance. The values of this factor ranged from −1.52 to 6.9; positive values of the symptoms were determined in 35.2% of respondents. The third factor that integrated the three symptoms that were related by their attributes to respiratory system problems was named the respiratory latent factor. It accounted for 14.76% of total variance; the values ranged from −2.05 to 6.09; positive symptoms were determined in 19% of respondents. The fourth factor that integrated the two symptoms which were related to the respondent's general well-being and loss of strength was named the asthenia latent factor. It accounted for at least −11.19% of the variance; the values ranged from −2.02 to 6.35; the positive values were determined in 26.5%.

The analysis of the factorial distribution of distress after balneotherapy treatment (Table 5) revealed that ten out of twelve symptoms met the conditions of the factorial analysis. Symptoms of anxiety and depression lost their influence on the distress risk; vomiting gained influence. During the analysis, the four latent factors that accounted for 59.24% of the total variance were distinguished. The factors were formed out of somewhat different groups of symptoms (several factors were regrouped). The priorities have changed over the course of the therapy, but we kept the same latent factor names based on the main symptom.

The first factor connecting the four symptoms that covered the symptoms related to the digestive system was attributed to the dyspeptic latent factor. It accounted for 17.06% of the total variance; factor values ranged from 0.85 to 9.13; positive values were determined for 28% of respondents. The second factor that integrated the two symptoms, which partially characterised the consequences of metal dysfunctions, was named mental. This distress latent factor accounted for 15.15% of the total variance. The values of factor ranged from −1.58 to 7.09; positive values of the symptoms were determined in 24% of respondents. The third factor that integrated the two symptoms related to respiratory system problems was named the respiratory latent factor. It accounted for 14.13% of the total variance; the values ranged from −1.49 to 4.45; positive symptoms were determined in 36% of respondents. The fourth factor has integrated the two symptoms which are more related to asthenia latent factor. It accounted for at least −12.9% of the variance; the values ranged from −1.44 to 6.13; the positive values were determined in 23.3%.

An assessment of the impact of the change in distress symptoms after a course of balneotherapy with geothermal water showed qualitative changes: the latent mental risk factor lost its total variance and moved to second place.

**Figure 2:**
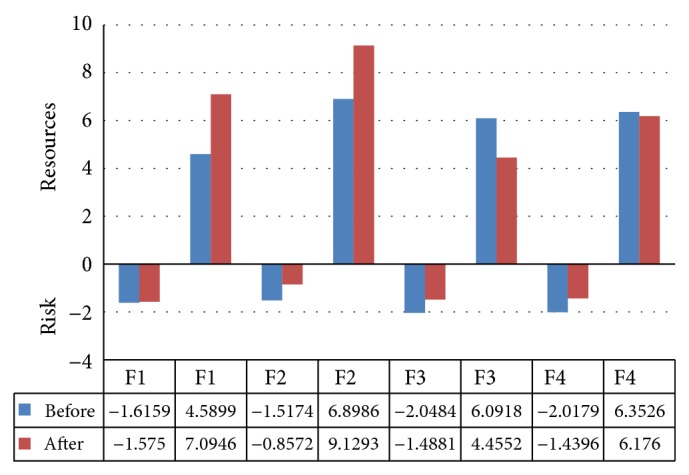
Factorial distribution of changes in distress for health resources and risks. ^*^F1: mental, F2: dyspeptic, F3: respiratory, F4: asthenic latent factors.

Balneotherapy's impact on distress-related health risks and resources was positive: it reduced health risk induced by all distress latent factors and increased resources induced by mental and dyspeptic factors. Resources induced by respiratory and asthenia factors were slightly reduced (Figure 2). As a result of the balneotherapy course, the overall distress health risk decreased by 26% (from 7.17 to 5.34), while the resources increased by 11% (from 23.93 to 26.85).

Tables 6 and 7 provide the general health overall risk situation before and after the balneotherapy treatment, including the distress-independent variables (the number of symptoms and the intensity of symptoms and their control). The odds ratio presented in column Exp(*B*) shows that the evolution of the risk possibility gains 1.

An evaluation of the results of the change in general health condition risks induced by distress shows that balneotherapy reliably reduced the probability of the risk of general health condition deterioration induced by the number of distress symptoms by 18% (before treatment, the probability of health deterioration was 1.802 times; after treatment, it was 1.475 times).

## 4. Discussion

Our clinical study has demonstrated the positive impact of balneotherapy with geothermal water on distress: the overall number of distress symptoms reduced by 60%, and their intensity by 41%, while the stress management has increased by almost 32%. A computer analysis showed the advantage in the geothermal group compared with the control group. We have received positive qualitative changes after balneotherapy: the explanatory variance of the latent mental factor decreased; anxiety and depression have lost value in risking health. Balneotherapy resulted in positive preventative results: the reduction of health risks and the growth of health recourses; the probability of distress-induced general health deterioration decreased by 18%. Our study showed the favourable effects of balneotherapy using geothermal water on pain, sleep disturbances, intestinal problems, loss of appetite, and shortness of breath (decreased factorial weight). After the treatment, the explanatory variance of all the latent factors was greater; that is, other systems' dysfunctions have shown potentially less impact after the therapy (cardiovascular, sensomotoric systems, and so forth, possibly improved). Perhaps a longer intervention would contribute to the reduction of other symptoms even more significantly.

According to the research data, the most significant distress symptoms in seafarers are lack of appetite, intestinal problems, and anxiety; four latent factors have been forming the manifestation of stress in the representatives of seafarers' lifestyle. The most important health risk factor for the seafarers was the mental factor, followed by dyspeptic, respiratory, and asthenia factors with certain attributes characteristic to each. In other words, the stress of seafarers can be identified and evaluated using anxiety, depression, and sleep scales. It would be advisable to measure these symptoms periodically (during a yearly health inspection). This research has shown that distress symptoms selected in GSDS reflect the larger part of the distress symptoms forming health risks. Established common latent distress risk factors of seafarers may be used in diagnostics or evaluations of the effects of treatment. These results could help in preparing rehabilitation programs for seafarers and other employees experiencing high levels of stress at work. It is advisable to improve the psychological environment, avoid conflicts, improve working conditions, reduce requirements, apply stress management strategies, and use recreational measures more frequently for saving human resources.

Our study method is used in the research of the effect of balneotherapy for the first time. Therefore, we cannot compare in terms of size of geothermal water baths effect. The influence of balneotherapy on health risks induced by stress has not been studied. Generally speaking, due to the heterogeneity of the balneotherapy study designs, methodological flaws, and the publication bias, a definitive conclusion about mineral water therapeutic power is not possible to state. The majority of the published papers focus on balneotherapy for musculoskeletal disorders [40, 44, 45, 48, 50–53], although an increasing number of publications report its application in other clinical fields: the drug sparing effect of balneotherapy can be utilized in psychiatry [63]; it is also useful in cardiovascular rehabilitation [64, 65], dermatology [66], and treatment of other diseases.

Based on the numerous Hungarian and non-Hungarian tap water-controlled studies, it can be stated that thermal mineral water alleviates pain caused by different musculoskeletal diseases regardless of the qualitative and quantitative composition of the mineral water; minerals might act on skin nerve endings and thereby achieve long-lasting gate control [48]. In comparison with tap water, treatment with alum-containing water demonstrated significantly greater progress, as reflected by the relief of pain elicited by handling the uterus and improvement of psychic status [49]. The duration of improvement after balneotherapy varies considerably, from two-three weeks to 40 weeks and even one year [25, 27, 48, 50–53].

According to M. Vitale on the basis of PubMed data, most of the researches on the effects of balneotherapy in the period from 2000 to 2013 were devoted to inflammation and skin related issues, while little of the research involved the nervous system and cell apoptosis [28]. To this day, there is inadequate evidence for the use of mineral water for psychoneurological diseases. Research of D. Marazziti and others emphasizes a marked effect of balneotherapy on the production of serotonin in blood, which improves the patient's psychoemotional state and relieves depression [67]. Ch. F. Roques' review discusses the effect of balneotherapy on generalised anxiety (50% improvement or recovery, the amount of the effect: 0.75, and duration: 6 months) [68], which is complemented by the studies of Dubois et al. [63]; Xiu and coauthors have proven mineral water to improve the state of mind in pilots: decreased scales of tension, anger, fatigue, and confusion (*P* < 0.05) [37]; an average impact of balneotherapy on stress reduction was demonstrated by Japanese scientists [20], whereas Blasche et al.'s study on pilots showed reduction in the burnout symptoms [69].

Lithuania, compared to its neighbouring countries, is distinguished by favourable geothermal conditions, particularly in the western part of the country. The most promising places in terms of balneology due to high temperature and mineralisation are the Lower Devonian and the Cambrian aquifers [70], from which resources can be used for balneotherapy to reduce the population's health risks and increase health resources.

The medicine of the 21st century is predicted to be dominated by such preventive activities. This may be a great chance and a very important message for future spas.

## 5. Conclusions

1. Balneotherapy reduces health risks by neutralizing the negative effect of factors of dyspeptic, respiratory, and asthenic nature and by reducing the number of distress symptoms.
2. Balneotherapy, using high mineralisation geothermal water, seems to be an effective preventative tool for managing the likelihood of health risks and increasing health resources.
3. Balneotherapy is a safe, easily accessed nonpharmacological method of regulating the body autonomously, which could have a significant place in integrative medicine once more widely studied and standardised.

## Figures and Tables

**Table 1:** The mineral composition of geothermal water.

Element	Concentration, mg/L
Cl^−^	66930
Na^+^	27580
Ca^2+^	8990
Mg^2+^	2630
SO_4_^2−^	1330
K^+^	690
HCO_3_^−^	74
Br	60.62
N	22
Fe	12.14
B	6.501
Si^4+^	4.886
Li^+^	1.200
Cr	1
F^−^	0.91
Mn^2+^	0.501
H_2_S	0.33
Cu^2+^	0.167
Zn^2+^	0.062

Total amount of dissolved mineral substances, mg/L	108224

**Table 2:** Demographic and basic clinical characteristics of the groups.

Sociodemographic characteristics	Geothermal group *N* = 65	Control group *N* = 65
Age, average (SD)	46.5 (10.6)	46.2 (9.3)
Period of service, average (SD)	22.5 (11.4)	22.4 (9.9)
Health-related factors		
BMI, average (SD)	27.1 (2.9)	28.7 (5.1)
Morbidity, *N* (%)	59 (92.2)	34 (68.0)
Use of medication, *N* (%)	19 (29.7)	23 (46.9)
Good subjective health condition, *N* (%)	33 (50.8)	27 (54.0)
Smoking, *N* (%)	27 (42.2)	24 (48)
Alcohol at work once in several weeks, *N* (%)	8 (12.9)	1 (2.0)
Frequent stress, *N* (%)	16 (24.6)	12 (24.5)
Stress intensity, VAS, average (SD)	3.9 (1.6)	3.6 (1.7)
Frequent pain, *N* (%)	4 (6.2)	3 (6.0)
Pain intensity, VAS, average (SD)	3.05 (1.6)	2.4 (1.6)
Fatigue intensity (7-point scale), average (SN)	3.4 (1.3)	3.3 (1.0)
Insufficient quality of sleep, *N* (%)	21 (32.3)	12 (24.5)

**Table 3:** Comparison of the effect on distress in the study's groups.

		Geothermal group *N* = 55		Control group *N* = 50	
		Average (SD)	*P* value	Average (SD)	*P* value
Number of symptoms	Before	4.35^**^ (1.85)	<0.001	3.32^**^ (1.77)	0.722
	After	1.71^***^ (1.38)		3.38^***^ (1.31)	

Intensity of symptoms	Before	5.41^***^ (1.78)	<0.001	3.82^***^ (1.83)	0.894
	After	3.16^*^ (1.95)		3.80^*^ (1.29)	

Control of symptoms	Before	5.64^*^ (1.99)	<0.001	6.44^*^ (2.05)	0.033
	After	7.62^***^ (2.21)		6.00^***^ (1.68)	

For comparisons, means before and after used paired-samples *t*-test.

For comparisons, means between geothermal and control groups used students *t*-test:

^*^ *P* < 0.05, ^**^ *P* < 0.01, and ^***^ *P* < 0.001.

**Table 4:** Factorial distribution of seafarers' distress symptoms before treatment.

Symptoms (descending order of factorial weights)	Factorial weight			
	(1) Mental	(2) Dyspeptic	(3) Respiratory	(4) Asthenia
Symptoms more related to factor 1				
Anxiety	0.806			
Depression	0.730			
Sleep disturbances	0.500	0.462		

Symptoms more related to factor 2				
Intestinal problems		0.865		
Pain		0.547		
Lack of concentration		0.499		

Symptoms more related to factor 3				
Shortness of breath			0.776	
Nausea			0.761	
Cough			0.481	

Symptoms more related to factor 4				
Lack of appetite				0.879
Fatigue				0.413

Eigen value	1.73	1.70	1.62	1.23
Percentage of the variance explained	15.75	15.52	14.76	11.19
Min	−1.6149	−1.5174	−2.0484	−2.0169
Max	4.5899	6.8986	6.0918	6.3526
Percentage of negative values	67.3	64.8	81.0	73.5
Percentage of positive values	32.7	35.2	19.0	26.5

**Table 5:** Factorial distribution of seafarers' distress symptoms after treatment.

Symptoms (descending order of factorial weights)	Factorial weight			
	(1) Dyspeptic	(2) Mental	(3) Respiratory	(4) Asthenia
Symptoms more related to factor 1				
Nausea	0.781			
Vomiting	0.719			
Pain	0.515	0.406		
Sleep disturbances	0.467			

Symptoms more related to factor 2				
Lack of concentration		0.833		
Intestinal problems		0.735		

Symptoms more related to factor 3				
Cough			0.836	
Fatigue			0.778	

Symptoms more related to factor 4				
Lack of appetite				0.832
Shortness of breath				0.651

Eigen value	1.70	1.51	1.41	1.29
Percentage of the variance explained	17.06	15.15	14.13	12.9
Min	−0.8572	−1.5750	−1.4881	−1.4396
Max	9.1293	7.0946	4.4552	6.1276
Percentage of negative values	72.0	76.0	64.0	73.7
Percentage of positive values	28.0	24.0	36.0	23.3

**Table 6:** Risk of general health condition affected by distress in seafarers before the treatment.

Independent variables^*^	*B*	S.E	Wald criterion	df	*P*	Exp(*B*)	95% GS, CI
GSDS_1_number	0.589	0.116	25.816	1	0.000	1.802	1.436–2.262
GSDS_1_intensity	−0.210	0.105	4.021	1	0.045	0.810	0.660–0.995
GSDS_1_control	−0.087	0.081	1.154	1	0.283	0.917	0.783–1.074
Constant	−0.959	0.806	1.416	1	0.234	0.383	

^*^GSDS: General Symptoms Distress Scale; number of stress symptoms, intensity, and control of stress symptoms.

**Table 7:** Risk of general health conditions affected by distress in seafarers after the treatment.

Independent variables^*^	*B*	S.E	Wald criterion	df	*P*	Exp(*B*)	95% GS, CI
GSDS_2_number	0.388	0.150	6.706	1	0.010	1.475	1.099–1.978
GSDS_2_intensity	−0.048	0.130	0.134	1	0.714	0.953	0.739–1.231
GSDS_2_control	−0.161	0.095	2.849	1	0.091	0.851	0.706–1.026
Constant	−0.079	0.929	0.007	1	0.932	0.924	

^*^GSDS: General Symptoms Distress Scale; number of stress symptoms, intensity, and control of stress symptoms.

## Methodological Strengths and Limitations

To our knowledge, this study is the first to explore the effects of geothermal mineral water of a very high concentration. We first used the integrated analysis of distress symptoms' influence on health risk and examined balneotherapy's effect on health risks, resources, and the probability of the evolution of general health conditions. The limitations of our study are the evaluation of the effects covering only one profession, the subjective measures of stress, the open-label, and no follow-up. Our study stimulates further research in this area.
